# The Prevalence, Predictors, and In-Hospital Mortality of Hepatic Encephalopathy in Patients with Liver Cirrhosis Admitted at St. Dominic Hospital in Akwatia, Ghana

**DOI:** 10.1155/2020/8816522

**Published:** 2020-12-22

**Authors:** Amoako Duah, Adwoa Agyei-Nkansah, Foster Osei-Poku, Francisca Duah, Daniel Ampofo-Boobi, Bright Peprah

**Affiliations:** ^1^Department of Medicine, St. Dominic Hospital, Akwatia, Ghana; ^2^Department of Medicine, University of Ghana Medical School, Korle-Bu Teaching Hospital, Accra, Ghana; ^3^Laboratory Department, Ga-North District Hospital, Ofankor, Accra, Ghana; ^4^Department of Biostatistics, St. Dominic Hospital, Akwatia, Ghana

## Abstract

**Background:**

Hepatic encephalopathy (HE) is one of the most debilitating complications of cirrhosis leading to death. Decrease in HE mortality and recurrence has been linked with timely identification and early treatment. There is a need to document the burden, predictors, and treatment outcomes of HE in an adult population with liver cirrhosis in our setting as only reports from resource-endowed countries abound in the literature. This study aimed therefore to determine the prevalence, predictors, and treatment outcomes of patients with liver cirrhosis admitted at St. Dominic Hospital (SDH) in Akwatia, Ghana.

**Materials and Methods:**

A prospective study was conducted involving one hundred and sixty-seven (167) patients admitted at the medical wards in SDH with liver cirrhosis from January 1st, 2018, to March 24th, 2020. The demographic and clinical features of the patients were collected using a standardized questionnaire. Biochemical, haematological, and abdominal ultrasound scans were done for all patients. Patients were then followed up until discharge or death.

**Results:**

There were 109 (65.3%) males out of the 167 patients with a mean age of 45.8 and 47.5 years for those with and without HE, respectively. The prevalence of HE was 31.7% (53/167). Out of 53 participants with HE, 75.5% (40/53) died. There was a strong association between HE and death (*p* < 0.001). The major precipitating factor of HE was infection (64.2%). Severe ascites (OR = 0.009) were clinical feature independently associated with HE, whereas high creatinine (OR = 0.987), blood urea nitrogen (BUN) (OR = 1.199), Child–Pugh score (CPS) (OR = 5.899), and low platelets (OR = 0.992) were the laboratory parameters and scores independently predictive of HE.

**Conclusion:**

HE was common among patients with liver cirrhosis admitted at SDH with high in-patient mortality. The commonest precipitating factor for HE was infection(s). Severe ascites, low platelet count, high creatinine, BUN, and CPS were independent predictors of HE.

## 1. Introduction

Hepatic encephalopathy (HE) is one of the most common complications of liver cirrhosis and is defined as a brain dysfunction caused by liver insufficiency and/or portosystemic shunts, resulting in significant impairment of the quality of life and frequent hospitalisations [1]. The cognitive impairment associated with HE severely affects the lives of patients and their caregivers. HE results in utilization of more healthcare resources in adults than other complications of liver disease [2]. HE diagnosis remains essentially clinical. Patients may present with progressive disorientation, inappropriate behaviour, and acute confusional state with agitation or somnolence, stupor, and eventually coma [1]. A wide spectrum of motor disorders can be observed in HE, including asterixis, hypertonia, hyperreflexia, and extrapyramidal dysfunction [1]. Unless the underlying liver disease is successfully treated, HE is associated with poor survival and a high risk of recurrence [3, 4]. The spectrum of HE ranges from minimal brain function deficits, known as minimal HE, to hepatic coma [5–7]. Overt HE occurs in approximately 30%–45% of patients with cirrhosis, while minimal HE may affect up to 60% of patients with chronic liver disease and up to 80% with cirrhosis [8–10]. However, the accurate data on the true incidence and prevalence of HE is lacking, mainly because of big differences in the aetiology and severity of HE and the difficulty in diagnosing minimal HE [11].

The classification of HE is based on the following four factors [1]: (1) according to the underlying disease, where HE is subdivided into type A resulting from acute liver failure, type B resulting predominantly from portosystemic bypass or shunting, and type C resulting from cirrhosis; (2) according to the severity of manifestations based on West Haven classifications; (3) according to its time course, where HE is subdivided into Episodic HE, Recurrent HE, and Persistent HE; (4) according to the existence of precipitating factors, where HE is subdivided into nonprecipitated or precipitated and the precipitating factors. Episodic HE is usually related to precipitating factors, such as infection, gastrointestinal bleeding, diuretic use, electrolyte disorder, and constipation [12].

The pathogenesis of HE in cirrhosis is complex and multifactorial, but the key role is thought to be played by circulating gut-derived toxins of the nitrogenous compound, most notable ammonia. Proper management of HE principally involves the recognition and control of these precipitant conditions, along with general and specific measures such as airway management, intensive care unit admission in severe cases, managing the complications of encephalopathy, reducing nitrogenous load in the gut, and assessing the need for long-term therapy and liver transplant evaluation [13]. The main goal of HE treatment is aimed at reducing the production of ammonia and maximizing the body's removal of ammonia from the bloodstream. Ammonia-lowering therapies such as nonabsorbable disaccharides (lactulose, lactitol, etc.) and selected antimicrobial (metronidazole, rifaximin etc.) are the main agents used to treat overt HE. In resource limited setting like Ghana, there is a dire need to document the burden, precipitating factors, and treatment outcomes of hepatic encephalopathy in the adult population with chronic liver disease to help develop a more appropriate management guideline taking cognizance of resource availability. This study aimed therefore to address this issue in patients with liver cirrhosis admitted at St. Dominic Hospital in Akwatia, Ghana.

## 2. Methods

### 2.1. Study Design

This was a prospective hospital-based study.

### 2.2. Study Area and Period

The study was carried out at the Department of Medicine, St. Dominic Hospital (SDH) in Akwatia, Ghana from January 1st, 2018, to March 24th, 2020. SDH is a 339-bed district hospital located in the Denkyembour district, Akwatia in the Eastern region of Ghana. It was founded in 1960 and serves as the main referral center for other surrounding district hospitals. It offers a breadth of medical and surgical services including gastroenterology, neurology, ophthalmology, obstetrics and gynecology, and endoscopy services.

### 2.3. Study Population and Sampling

The study population comprised adult patients with liver cirrhosis admitted to the medical unit of SDH during the study period. The sample size was determined using the Cochran formula for sample size calculation. With an estimated prevalence of 10% for liver cirrhosis (14), a *Z*-score at 95% confidence level (1.96), and a level of significance of 0.05, the minimum sample size was calculated to be 139. Due to the high attrition rate for prospective studies, 186 participants were consecutively recruited after meeting study criteria and giving informed consent. Diagnosis of liver cirrhosis was made based on the clinical features, laboratory investigations, and abdominal ultrasound findings suggestive of liver cirrhosis.

### 2.4. Inclusion Criteria

Inclusion criteria include all adult patients above 18 years with diagnosis of liver cirrhosis.

### 2.5. Exclusion Criteria

1. Patients with severe primary cardiopulmonary failure, intrinsic renal disease, and obstructive jaundice were excluded from the study
2. Patient with septic encephalopathy were excluded

### 2.6. Management of Hepatic Encephalopathy in Ghana

Management principles of decompensated liver cirrhosis at the study site and for Ghana in general are by identifying and management of precipitating factors and the underlying cause of the liver disease. These include detailed history, blood work, imaging, and endoscopy as needed.

Medical management of these patients includes the use of lactulose and oral metronidazole. Lactulose is given orally or via nasogastric tube aiming at two to three bowel movements per day. Available management of decompensated liver cirrhosis at the study site also pertains to Ghana in general is by identifying and treating complications leading to admission and managing the underlying cause of liver cirrhosis. Although rifaximin, L-ornithine-L-aspartate (LOLA), and flumazenil are known to be efficacious in the management of hepatic encephalopathy, it is generally not available and, in few areas, where these drugs are available, are beyond the pockets of most patients. For variceal bleeding patients were first stabilized with blood transfusion and/or intravenous fluids followed by variceal band ligation. Vasoactive drugs such as Octreotide and Terlipressin are available but not nationwide and are financially not accessible by all patients. Patients admitted with ascites are managed with diuretics mainly spironolactone and furosemide. Those with severe ascites compromise their respiration and refractory ascites, and abdominal paracentesis is done without albumin infusion in most patients because albumin is expensive and not covered by our national health insurance. This limits the volume of fluid drained per day. Transjugular intrahepatic portosystemic shunt (TIPS) and liver transplant services are not available in Ghana. None of the participants was managed in an intensive care unit irrespective of the grade of HE because intensive care unit (ICU) facility is not available at the study site and ICU space is limited in Ghana.

### 2.7. Data Collection and Measurements

After thoroughly explaining the study to patients, individuals who gave informed consent were recruited and a questionnaire was administered to obtain sociodemographic data and clinical history. Relevant histories including alcohol use and clinical features of liver cirrhosis (spider angioma, palmar erythema, ascites, asterixis, hepatomegaly, splenomegaly, and abdominal vein collaterals) were obtained. The other information collected includes treatment given to patients with HE and the outcome of their admission (discharge or dead). A diagnosis of hepatic encephalopathy was made when the patient had impaired consciousness with a background liver cirrhosis in the absence of any neurological disorder or other causes of impaired consciousness. The West Haven criterium was used in grading the encephalopathy [14]. It is a semiquantitative grading of mental state from trivial lack of awareness (grade 1) to coma (unresponsive to verbal or noxious stimuli (grade 4). The Child–Pugh scoring (CPS) system was used for assessing the severity of liver disease on patient presentation [15]. The scoring system takes into account the serum albumin, serum prothrombin time or international normalized ratio (INR), and bilirubin as well as the presence of fluid retention and encephalopathy, each of which is given a numerical score. There are 3 grades: A, B, and C depending on the total scores. Model for Endstage Liver Disease sodium (MELD-Na) was also calculated for all patients [15].

A sample of 15 mls of venous blood was taken for haematological, biochemical, and serological investigations. Abdominal paracentesis was performed using an aseptic technique at the right or left iliac fossa, 3 cm above, and 3 cm medial to the anterior superior iliac spine. Exactly 15 mls of ascitic fluid was collected using a sterile syringe for culture, cell count and differential, albumin, and protein. The diagnosis of spontaneous bacterial peritonitis was based on demonstration of more than 250 neutrophils/cm^3^ or positive fluid culture in ascitic fluid. Urine analysis (proteins, leucocytes, erythrocytes, pus cell, and other urine abnormalities) was done for all patients. All patients were tested for HBsAg and anti-HCV Ab to determine the cause of liver cirrhosis. Chest X-ray was done for all patients with clinical diagnosis of pneumonia, mainly to look for areas of consolidation. Furthermore, an abdominal ultrasound scan was performed for all patients. The following details were recorded: maximum vertical span of the liver; nodularity of liver surface; spleen size (length of its longest axis); and presence of ascites.

### 2.8. Ethical Approval and Informed Consent

Formal approval of this study was obtained from the institutional Ethical and Review Committee of the St. Dominic Hospital. This study was conducted in accordance with the Helsinki Declaration on Human Experimentation, Sixth Revision (October 2008). The nature of the study was fully explained to potential participants. It was explained that the study was entirely voluntary and that nonparticipation would not jeopardize their immediate or subsequent medical care at the hospital. Participants who agreed to participate were asked to sign an informed consent form. For those patients with HE, written consent was obtained from caregivers.

### 2.9. Statistical Analysis

The data obtained were analyzed using the statistical package for social sciences (IBM SPSS, version 23) statistical software. Descriptive statistics were undertaken for all the variables and data presented in appropriate tables. The causes of liver cirrhosis and the prevalence of HE were determined. Further analysis was done to determine if there were any associations between HE and the clinical or laboratory parameters. Chi-square was used to determine the level of association. A binary logistic regression analysis was conducted for clinical, laboratory parameters, and prognostic scores (CPS, MELD-Na) to determine if any of them were a predictor of HE. For all analyses, *p* values <0.05 were considered statistically significant.

## 3. Results

### 3.1. Demographic Characteristics and Treatment Outcome

A total of 167 patients were recruited, comprising of 109 (65.3%) males. Their ages ranged from 20 to 77 years with a median age of 45.8 and 47.5 years for those with and without HE, respectively. The prevalence of HE was 31.7% (53/167). Out of 53 participants with HE, 75.5% (40/53) died, contributing 60.6% of all in patient mortality among participants. There was a strong association between HE and death (*p* ≤ 0.001). The length of stay and blood pressure of the participants is shown in Table 1.

### 3.2. Grades of HE

The majority of patients admitted had HE Grade 3, 37.7% (20/53) and Grade 4, 35.8% (19/53) severity (Table 2).

### 3.3. Causes of Liver Cirrhosis

The cause of liver cirrhosis was significant alcohol consumption and hepatitis B virus in 75 (44.9%) and 73 (43.7%) of participants, respectively. Other causes were hepatitis C virus in 10 (6.0%), nonalcoholic liver disease (NAFLDx) in 5 (3.0%), and schistosomiasis 4 (2.4%). In 13 (7.8%) of the patients, causes of their liver cirrhosis were not identified (Table 3).

### 3.4. Precipitating Factors of HE

The major precipitating factors of HE were infection (34/53, 64.2%) [pneumonia (24.5%), urinary tract infection (17.0%), spontaneous bacterial peritonitis (13.2%), cellulitis (5.7%), sepsis of unknown source (3.8%)], gastrointestinal bleeding (7/53, 13.2%), and electrolyte imbalance (10/53, 18.9%) [hypokaelemia (6/53, 11.4%) and hyponatremia (4/53, 7.5%)]. Dehydration and constipation accounted for (4/53, 7.5%) and (2/53, 3.8%), respectively. Unknown precipitants accounted for (7/53, 13.2%) (Table 4).

### 3.5. Clinical Features and Laboratory Parameters

There was no clinical feature significantly associated with HE. However, there was a significant association of those admitted without HE and weight loss (*p* < 0.009). The laboratory parameters with significant association with HE were elevated bilirubin, INR, creatinine, BUN and gamma glutamyl transferase (GGT), and low serum albumin, platelet count, and sodium. High CPS and MELD-Na also had significant association with HE (Tables 5 and 6).

### 3.6. Logistic Regression of Independent Variables Associated with Encephalopathy

Severe ascites was clinical feature independently associated with HE. Elevated creatinine, blood urea nitrogen (BUN), low platelets, and high CPS were the laboratory parameters and score that were independently predictive of HE (Table 7).

## 4. Discussion

Hepatic encephalopathy is one of the most common complications of cirrhosis, and it has been associated with a significant impact on patients' health-related quality of life and survival, independently of the severity of cirrhosis [13]. HE management involves excluding other causes of altered mental status, caring for the unconscious patient, determining, treating precipitating factors, and initiating empiric therapy [16]. This study aimed therefore to determine the prevalence, precipitating factors, and treatment outcomes of patients with liver cirrhosis admitted at St. Dominic Hospital in Akwatia, Ghana. HE at admission was observed in 31.7% of patients included in this study. This number is similar to that reported by the CANONIC (34%) and NACSELD (33%) cohorts [17, 18] but lower than that reported by Alexopoulou et al. [19]. These differences are probably explained by the specific characteristics of the cohorts (differences in the aetiology and severity of HE) and difficulty in diagnosing minimal HE and by the fact that patients were evaluated very early after admission in this study. The high mortality in our patients may be multifactorial. Important medications needed in treating these patients such as Rifaximin, LOLA, flumazenil, octreotide, and terlipressin were either not available or where available patients could not afford to buy them because they are not on health insurance. Some baseline investigations were also difficult to do due to financial challenges. Procedures and interventions like TIPSS and liver transplant services are not available in the country. Seriously ill patients requiring admissions to the high dependency unit (HDU) or the ICU were managed on the main ward because such facilities do not exist in the study site and even in areas where they exist are few and always over prescribed.

Studies show that malnutrition and muscle mass loss (sarcopenia), which has often been used as an equivalent of severe malnutrition [20], is associated with a higher rate of HE [21]. Among cirrhotic patients, 75% of those who develop HE have moderate to severe malnutrition, which affects their energy reserves and muscle mass. Elsaid and Rustgi [22] identified sarcopenia as a risk factor for developing HE. Due to the muscular involvement in ammonia metabolism, malnutrition is associated with a higher incidence of HE [23]. Under normal physiological conditions, ammonia is metabolized by the liver, brain, muscle, and kidney. In cirrhotic patients, the affected liver has an impaired capacity for removal of ammonia in the form of urea, which may result in increased muscle glutamine synthetase to provide an alternative mechanism for ammonia removal as glutamine. In malnourished cirrhotic patients, the loss of muscle mass, commonly seen as a consequence of malnutrition, can adversely affect this alternative route of ammonia removal leading to HE development. In the current study, though weight loss was not an independent predictor of HE, but 90.6% (48/53) of the patients with HE presented with weight loss. Though in this study weight loss was not a predictor of HE and the reason for this may be due to the higher number of patients who presented with weight loss and only 31.7% had HE among the participants. Ascites is a clinical manifestation of decompensated liver cirrhosis and one of the clinical components of the CPS, an established measure of severity of liver disease. HE tends to be higher in patients with decompensated liver cirrhosis compared to compensated liver cirrhosis [1].

Patients who developed HE in this study had laboratory values suggesting more advanced liver disease: highly elevated bilirubin and INR and lower albumin, platelet, and sodium values. At the same time, CPS and MELDNa were higher in patients with HE compared to those without. Elevated creatinine and BUN, low platelets, and high CPS were laboratory parameters and scores that were independent predictors of HE. Albumin and bilirubin comprise 2 of 3 objective components of the CPS, an established measure of severity of liver disease [15]. Bai et al. in their study concluded that decreased serum albumin level may be associated with higher risk of overt HE and HE-associated mortality during hospitalisations in patients with liver cirrhosis [24]. Other studies have also shown that albumin infusion might prevent the occurrence of overt HE and improve the severity of overt HE [25]. Kamath et al. [26] reported that laboratory predictors associated with HE were albumin, total bilirubin, INR, creatinine, sodium, platelet count, and transformed variables such as the model for end-stage liver disease. Guevara et al. [27] in their study also identified hyponatremia, serum bilirubin, and serum creatinine as an independent predictive factor of overt HE. Other studies have also reported hyponatremia, high creatinine, high bilirubin, and low albumin as risk factors for overt HE [22]. The incidence of HE was the highest among those with portal hypertension. According to Tapper et al. [28], low platelet is an indicator of advanced liver disease and an indication of portal hypertension.

The major precipitants encountered in the current study were infections, electrolyte abnormalities, and gastrointestinal bleeding which were in line with other studies conducted in Nigeria [29]. Infections, constipation, and gastrointestinal bleeding were identified as the major precipitants of HE in the study conducted by Mumtaz et al. [30], in Pakistan. Commonly observed precipitating factors of HE by Raphael et al. [31] were the use of diuretics in massive ascites, infections, and GI bleeding. Literature from developed countries, however, has not identified infections among the most common precipitating events [32]; this probably reflects the hygienic, nutritional, and immune status in their patients. Patients in the local settings are usually severely malnourished not only because of their disease but also because of food faddism and taboos regarding their diet [33]. Strict dietary restrictions on these patients lead to anorexia and malnutrition and eventually lowering their immunity and making them more susceptible to infections. Other reasons for the difference in the precipitating factors from different patients from various countries may be as a result of the demographic characteristics of the patients in terms of aetiology and severity of liver disease, and the drugs (diuretics, benzodiazepines, lactulose or lactitol, etc.) the patients were taking before or during the studies.

The development of HE is associated with a poor outcome. HE typically heralds hepatic decompensation in patients with chronic liver disease, and its development is usually associated with high mortality, indicating the need for liver transplantation. In-hospital mortality rate of patients with HE in this study was 75.5%. This is similar to 75% reported by Raphael et al. [31], in their study conducted in Tanzania. However, this is higher than 48% reported in a study conducted in Nigeria [29] and 33.3% reported by Udayakumar et al. in India [34]. Sasidharan et al. [35] also reported in-hospital mortality rate of 69.5% in a study conducted in India which is also lower than 75.5% reported in this study. The differences in mortality rate may be due to the variations in the underlying aetiology, severity of HE, precipitating factors, and resources available for patient's management. In addition, other underlying comorbidities of the patients could have also contributed to the difference in in-hospital mortality rates. The majority of patients had West Haven classification grades 3 and 4 HE in this study. Lactulose and metronidazole were the main drugs used for the treatment of HE in this study in contrast to more superior and potent drugs such as rifaximin which are not available in our setting. None of the participants also had HDU or ICU care because of nonavailability at the study site. Furthermore, late presentation, inaccessibility of definite therapies (liver transplants and TIPS), and advanced hepatology centers in Ghana could also have contributed to the differences observed.

### 4.1. Limitations

This study was not without limitations. Due to the high cost of neuroimaging patient, this could not be carried out to exclude primary neurological diseases that could mimic HE. However, most of these patients already had preexisting liver disease.

## 5. Conclusion

HE was common among patients with liver cirrhosis admitted at SDH with high in-patient mortality. The commonest precipitating factors for HE were infection(s), more especially pneumonia, UTI, and SBP. Severe ascites, low platelet count, high creatinine, BUN, and CPS were independent predictors of HE. Late presentation of these patients poses challenges in management and survival. Early screening of all patients with liver cirrhosis, especially those at high risk of HE, should be done to diagnose subclinical HE, enabling early identification of precipitating factors, so that appropriate treatment could be instituted. Other potent drugs such as rifaximin and more intensive care units should be made available in this country to reduce the mortality rate of HE.

## Figures and Tables

**Table 1:** Demographic characteristics, length of stay, and treatment outcomes against encephalopathy.

Demographic	Encephalopathy		
	Present	Absent	*p* value
Age of patient	45.8 (34.5, 57.1)	47.5 (33.7, 61)	0.448
Sex of patients			0.51
Female	24 (41.4%)	34 (58.6%)	
Male	29 (26.6%)	80 (73.4%)	
Blood pressure (BP)			
Systolic BP	109.3 (93.5, 123.1)	107.6 (93.7, 121.5)	0.753
Diastolic BP	69.6 (60, 79.2)	68.8 (58.5, 79.1)	0.62
Length of stay	11.51 (4.4, 18.6)	9.95 (3.9, 16.02)	0.145
Outcome of admission			**<0.001**
Death	40 (60.6)	26 (39.4)	
Discharge	13 (13.3)	85 (86.7)	

**Table 2:** Severity of hepatic encephalopathy.

Severity of encephalopathy (*n* = 53)	Frequency	Percentage
Grade 1	0.0	0.0
Grade 2	14	26.4
Grade 3	20	37.7
Grade 4	19	35.9
Total	53	100.0

**Table 3:** Causes of liver cirrhosis.

Aetiology	Number	Percentage
Alcohol	75	44.9
Hepatitis B virus	73	43.7
Hepatitis C virus	10	6.0
NAFLDx	5	3.0
Schistosomiasis	4	2.4
Unknown	13	7.8

Multiple response analysis (total >167).

**Table 4:** Precipitating factors for hepatic encephalopathy.

Precipitants	Frequency	Percentage
Infections	34	64.2
*Pneumonia*	13	24.5
*Urinary tract infection*	9	17.0
*Spontaneous bacteria peritonitis*	7	13.2
*Cellulitis*	3	5.7
*Sepsis*	2	3.8
Electrolyte abnormalities	10	18.9
*Hypokaelemia*	6	11.4
*Hyponatraemia*	4	7.5
Gastrointestinal bleeding	7	13.2
Dehydration	4	7.5
Constipation	2	3.8
Unknown	7	13.2

Multiple response analysis (total >53).

**Table 5:** Clinical features of the study participants.

Clinical symptoms and signs	Encephalopathy (present)	Encephalopathy (absent)	Total	*p* value
	*N* (%)			
Ascites	47 (34.3)	90 (65.7)	137	0.055
None	6 (20.0)	24 (80.0)	30	
Mild	3 (50.0)	3 (50.0)	6	
Moderate	22 (45.8)	26 (54.2)	48	
Severe	21 (25.3)	62 (74.7)	83	
Abdominal pain	17 (27.9)	44 (72.1)	61	0.415
Fever	14 (43.8)	18 (56.2)	32	0.104
Chills	10 (50.0)	10 (50.0)	20	0.061
Weight loss	48 (36.6)	83 (63.4)	131	**0.009**
Hematemesis	10 (43.5)	13 (56.5)	23	0.193

**Table 6:** Laboratory parameters of the study participants.

Liver function	Encephalopathy (present) (*n* = 53)	Encephalopathy (absent) (*n* = 114)	*p* value
AST (U/L)	**142.7** (4.7, 280.7)	**109.7** (25.7, 193.7)	0.067
ALT (U/L)	**72.5** (10.7, 134.3)	**61.8** (16, 107)	0.211
ALP (U/L)	**471.2** (2.3, 940.1)	**384.6** (13.1, 756.1)	0.240
GGT (U/L)	**336.7** (4.5, 668.9)	**231.6** (7, 456.2)	**0.026**
Total bilirubin (*μ*mol/l)	**150.3** (5.2, 295.4)	**82.6** (−43.3, 208.5)	**0.002**
Total protein (g/l)	**69.3** (54, 84.4)	**69.3** (53, 85.3)	0.959
Serum albumin (g/l)	25.5 (18, 32.9)	29.4 (21.5, 37.4)	**0.003**
INR	3 (1.8, 4.2)	**2.1** (1.4, 2.8)	**<0.001**
CPS	12 (9.9, 14.3)	9.4 (7.3, 11.5)	**< 0.001**
MELD-Na	31 (23.5, 38.5)	22 (15.2, 28.8)	**<0.001**
Full blood count			
Haemoglobin (g/dl)	9.3 (1.8, 15.6)	10.2 (2.3, 17.2)	0.053
White blood cell (10^9^/l)	10.9 (1.3, 38.2)	13 (1.6, 438)	0.717
Platelet count (10^9^/l)	130.6 (7, 515)	176.3 (5.90, 705)	**0.015**
Renal function test			
Sodium (mmol/l)	130.4 (97.1, 146.7)	134 (118, 151)	**<0.001**
Potassium (mmol/l)	3.9 (2.1, 6.4)	4.1 (2, 7.2)	0.313
BUN (*μ*mol/l)	11.3 (1.6, 21)	7.2 (−1.0, 15.4)	**0.006**
Creatinine (*μ*mol/l)	157.9 (22.7, 625.3)	108 (21.4, 815.8)	**0.010**

ALT—alanine aminotransferase; ALP—alkaline phosphatase; GGT—gamma glutamyl transferase; AST—aspartate aminotransferase; BUN—blood urea nitrogen; MELD-Na—model for end-stage liver disease sodium; INR—international normalized ration; CPS—Child–Pugh score.

**Table 7:** Logistic regression of independent variables associated with encephalopathy.

Variables	*B*	SE	Wald	*p* value	Odds ratio	95% CI	
						Lower	Upper
Severe ascites	−4.722	1.445	10.677	**0.001**	0.009	0.001	0.151
Weight loss	−3.946	1.374	8.252	**0.004**	0.019	0.001	0.285
Total bilirubin (*μ*mol/l)	0.002	0.011	0.034	0.855	1.002	0.981	1.023
Serum albumin (g/l)	0.048	0.058	0.705	0.401	1.05	0.937	1.175
INR	−0.421	0.613	0.472	0.492	0.656	0.197	2.182
Sodium (mmol/l)	−0.078	0.079	0.968	0.325	0.925	0.791	1.081
Creatinine (*μ*mol/l)	−0.013	0.006	4.275	**0.039**	0.987	0.975	0.999
Haemoglobin (g/l)	−0.053	0.136	0.152	0.697	0.948	0.726	1.238
MELDNA	0.106	0.132	0.643	0.422	1.112	0.858	1.44
BUN (*μ*mol/l)	0.182	0.076	5.693	**0.017**	1.199	1.033	1.392
GGT	0.001	0.001	0.215	0.643	1.001	0.998	1.003
Platelet count (10^9^/l)	−0.008	0.003	4.865	**0.027**	0.992	0.986	0.999
CPS	1.775	0.424	17.521	**<0.001**	5.899	2.57	13.541
Constant	−9.87	12.588	0.615	0.433	0		

GGT—gamma glutamyl transferase; AST—aspartate aminotransferase; BUN—blood urea nitrogen; MELD-Na—model for end-stage liver disease sodium; INR—international normalized ration; CPS—Child–Pugh score.

## Data Availability

The data used to support the findings of this study are available from the corresponding author upon request.
